# Diabetes alters the expression of partial vasoactivators in cerebral vascular disease susceptible regions of the diabetic rat

**DOI:** 10.1186/1758-5996-5-63

**Published:** 2013-10-21

**Authors:** Renshi Xu, Rongwei Yang, Huoyou Hu, Qiujiang Xi, Hui Wan, Yuchen Wu

**Affiliations:** 1 Department of Neurology, the First Affiliated Hospital of Nanchang University, Nanchang, Jiangxi 330006, China

**Keywords:** Diabetes, Endothelial dysfunction, Endothelial activators, Cerebral vascular disease

## Abstract

**Background:**

The pathogenesis between cerebral vascular disease (CVD) and the endothelial dysfunction (ETD) remains elusive in diabetes. Therefore, we investigated the expression of partial vasoactivators which be closely relative to ETD in CVD susceptible brain regions in the diabetic rat. The aim was to search some possible pathogenesis.

**Methods:**

Diabetes was induced by a single intraperitoneal injection of streptozotocin and a high lipid/sugar diet. The expression of vasoactivators ET-1, CGRP, VCAM-1, ICAM-1 and P-selectin were assessed by immunohistochemical staining and measurement of optic density of positive cells in the frontal and temporal lobe, basal ganglia and thalamus at 4 weeks after establishment of the diabetic model.

**Results:**

The expression of ET-1, VCAM-1, ICAM-1 and P-selectin significantly increased and CGRP significantly decreased in the diabetic group, and the expression of these vasoactivators was significantly different among the frontal, temporal lobe, basal ganglia and thalamus, and among the emotion, splanchno-motor and neuroendocrine center in the diabetic group.

**Conclusions:**

Diabetes alters the expression of partial vasoactivators in cerebral vascular disease susceptible regions of the diabetic rat. Therefore, we suggested that CVD complications in diabetes are partly caused by ETD via an imbalance expression of endothelial vasoactivators, which might be associated with dysfunction of emotion, autonomic nerve and endocrine center. However, further studies are warranted.

## Background

The diabetes epidemic continues to grow. In the year 2000, there was an estimated 171 million patients worldwide with a diabetes diagnosis. This number is projected to rise to 366 million by 2030, with 90% having a diagnosis of type 2 diabetes (T2DM) [1]. T2DM is more common in people older than 45 who are overweight. It is a major risk factor for cerebral vascular diseases (CVD) and is associated with accelerated atherosclerosis and a high rate of arterial thrombotic complications. Patients with T2DM have a higher incidence of stroke and a poorer prognosis after stroke. T2DM is associated with both micro- and macrovascular complications that can lead to significantly elevated incidence of CVD and is frequently associated with atherosclerotic vascular disease involving CVD. A number of studies support the concept that endothelial dysfunction (ETD) contributes to the pathogenesis and progression of the vascular complications in diabetes [2-6].

ETD plays a key role in the initiation of cellular events evolving into the development of vascular complications in diabetes, and it is regarded as an important factor in the pathogenesis of vascular disease in obesity-related T2DM [3]. The abnormal production of endothelium-associated factors/vasoactivators including vascular oxidative stress [4], inflammatory reaction, nitric oxide (NO), prostanoids (prostacyclin), endothelin-1 (ET-1), angiotensin II (ANG-II), tissue-type plasminogen activator (t-PA), plasminogen activator inhibitor-1 (PAI-1), von Willebrand factor (vWF), adhesion molecules (Vascular cell adhesion molecule, VCAM; Leukocyte adhesion molecule; Intercellular adhesion molecule, ICAM), and cytokines leads to ETD [3]. This results in elevating vascular tone, which contributes to microvascular and macrovascular damages and apoptosis of microvascular cells, ultimately leading to diabetes-related vascular complications [3].

In a variety of pathological circumstances, the balance of these regulatory mediators is altered, resulting in the onset and progression of vascular ETD [5]. Vascular ETD increases interactions with leukocytes, smooth muscle growth, vasoconstriction, impaired coagulation, vascular inflammation, thrombosis, and atherosclerosis, which are the basis of most late diabetic complications such as retinopathy, nephropathy, vasculopathy and neuropathy [6].

The pathogenesis of diabetic vasculopathy in T2DM is very complicated due to the many independent factors involved. The relationship of diabetic CVD complications and the ETD-associated factors mentioned above is not completely understood, despite extensive research [2-6]. And there are fewer reports on ET-1, calcitonin gene related peptide (CGRP), VCAM-1, ICAM-1 and P-selectin expression in the CVD susceptible brain regions of diabetic rats. Moreover, we observed that these vasoactivators extensively expressed in the rat brain in our previous experimental results (The data no published). Therefore, in this study, we will further investigate the alterations of individual endothelial function-associated factors like ET-1, CGRP, VCAM, ICAM-1 and P-selectin in the susceptible brain regions of CVD such as frontal and temporal cerebral cortex, basal ganglia and thalamus (including the hypothalamus) in a rat model of T2DM. The aim is to search the applicable optimal candidate methods for specific therapies targeting these factors using moderators of the endothelial function system, which might assist to reverse vascular ETD and thus reduce the morbidity and mortality of the related CVD in diabetes. The results from our studies indicate that ET-1, VCAM-1, ICAM-1, P-selectin and CGRP expression in the partial frontal and temporal cortex, basal ganglia, nuclei of hypothalamus and the inferior part of the thalamus is significantly altered in the diabetic rat model. It might be of importance to retrieve the imbalance of ET-1, VCAM-1, ICAM-1, P-selectin and CGRP expression in the prevention of complications related to diabetic CVD.

## Methods

### Diabetic rat model

Sprague–Dawley rats were made diabetes by a single intraperitoneal injection of 0.1 mmol/L STZ buffer solution (STZ, 30 mg/kg body weight, Sigma Co. Ltd., USA) and a high lipid/sugar diet (common animal feed 66.5%, sugar 20%, lard 10%, cholesterol 2.5%, sodium cholate 1%) [7,8]. Weight- and age-matched control rats were injected with an equal volume of citric acid buffer solution and fed a routine diet. The blood of rat tail vein was taken for measure of blood glucose and insulin levels at baseline and the endpoint after 4 weeks of streptozotocin intraperitoneal injection. Only STZ-injected rats with above 15 mM of a fasting blood glucose level at endpoint were included in this study as the diabetic rats. Body weight, insulin levels and insulin sensitivity were monitored. Food and fluid intake as well as urine production were evaluated at baseline and after 4 weeks. Four weeks after the induction of diabetes, according to Longa’s standard of 5 levels of 4 scores, rats that received scores of 0 were allowed into the following experiments. The diabetic rats were sacrificed, and brain tissues were processed for experimental analysis. All rats in our experiment were male, the average age in the endpoint of experiment was 14.2 month. Each group consisted of 10 rats. All animal studies were conducted in accordance with the Guide for the Care and Use of Laboratory Animals of China. All experiments involving rats were reviewed and approved by the ethics committee for animal care and use of the First Affiliated Hospital of Nanchang University, China (Permit nr: 8/21.01.2009).

### Blood glucose, insulin levels and insulin sensitivity index measurement

The rat vein blood for blood glucose and insulin level measurements was drawn after 12 hours of fasting. Blood glucose levels were measured by Roche glucometer (Roche Group, Switzerland). Insulin levels were measured by radioimmunoassay with a kit (Shandong Weifang 3 V Bioengineering Group. Ltd., China). Insulin sensitivity index (ISI) was determined by the following formula: ISI = 1/(Blood glucose level + Insulin level).

### Immunohistochemical staining

Control and diabetic rats were anesthetised and perfused with 200 ml 0.9% cold saline and 400 ml 4% PFA in 1xPBS (pH 7.5) at room temperature. Rat brains were excised and placed in 4% PFA buffer overnight, followed by incubation in 20% sucrose in 1×PBS (pH 7.5) for 3 days. The brains were then embedded. The tissue was coronally sectioned using successive 30 μm cuts on a cryostat from the occipital to frontal lobe and collected on slides. For immunohistochemical staining, sections were permeated with 0.2% Triton X-100 and blocked with 10% goat serum in 1xPBS after being rehydrated in 1xPBS (pH 7.4). Sections were then incubated at 4°C overnight with the following antibodies: ET-1, CGRP (Sigma Co. Ltd.), VCAM-1, ICAM-1 and P-selectin (Sigma Co. Ltd.). All antibodies were diluted 1:50. Sections were then mildly washed 6 times with 0.2% Triton X-100 in 1×PBS for 5 minutes. Incubated with secondary antibody diluted 1:200 for 2 hours at room temperature(RT), extensively washed 3 times with 1xPBS for 5 minutes, and incubated with ABC compounds diluted 1:100 for 2 hours at RT, extensively washed 3 times with 1×PBS for 5 minutes. Stained with 0.03% DAB and 0.006% H_2_O_2_ in Tris HCl buffer solution (TBS, pH7.6) for 10 minutes at RT. Wash with ddH_2_O, counterstained with hematoxylin for 2 minutes, routinely dehydrated with alcohol, mounted with resin and examined under a light microscope with a spot digital camera. All immunohistochemical staining sections were observed, and pictures were taken.

### Image collection and analysis

All images were collected with a light microscope with a digital camera and analysed with image analysis software. For semi-quantification of positive cells, the images were displayed on a computer monitor at 20× magnifications. Optic density (OD) of the positive cells was measured with Image-Pro Plus 6.0 software (Media Cybernetics Co. Ltd., USA). Fifty positive cells from each distinct brain region were randomly chosen, 10 sections per animal with 10 rats per group. The OD of total positive cells for each chosen brain region was calculated. The average OD of each region was used for comparing the differences between the control and diabetic group. The average OD difference (Average OD difference = average diabetic OD - average control OD) was used to compare and analyze the rank order (from more to fewer) of the expression quantity change of ET-1, CGRP, VCAM-1, ICAM-1 and P-selectin in distinct brain regions.

### Statistical analysis

Statistical analysis of the data was performed using a Student’s t-tests. All data are presented as means ± SD. p < 0.05 was considered to be statistically significant.

## Results

### Baseline and endpoint characteristics in the control and diabetic rat

Table 1 presents baseline and endpoint characteristics of both nondiabetic (Control group) and diabetic rats (Diabetic group). On the comparison of baseline and endpoint characteristics between control and diabetic rats, the diabetic rats showed an approximately five-fold increase in blood glucose levels, decreased weight gain and decreased insulin sensitivity, also showed a increased insulin level, increased food and fluid intake and increased urine production at endpoint. The baseline and endpoint characteristics besides the physiological weight gain didn’t show any statistical difference in nondiabetic rats (Table 1).

**Table 1:** Comparison of baseline and endpoint characteristics in the control and diabetic rats

**Characteristics**	**Nondiabetic rats**		**Diabetic rats**	
	**Baseline**	**Endpoint**	**Baseline**	**Endpoint**
Blood glucose (mmol/L)	4.46 ± 0.39	4.70 ± 0.59	4.55 ± 0.23	20.9 ± 0.83*
Weight (g)	440.53 ± 10.33	498.55 ± 10.5	442 ± 9.66	390 ± 10.23*
Blood insulin (mU/L)	3.3 ± 0.24	3.25 ± 0.28	4.23 ± 0.37	77.16 ± 7.78*
Insulin sensitivity index	0.12 ± 0.05	0.13 ± 0.03	0.14 ± 0.02	0.02 ± 0.002*

*P < 0.05, Compared with baseline.

### Expression of ET-1 significantly increased in partial cerebral cortex, basal ganglia and thalamus of diabetic rats

**Figure 1:**
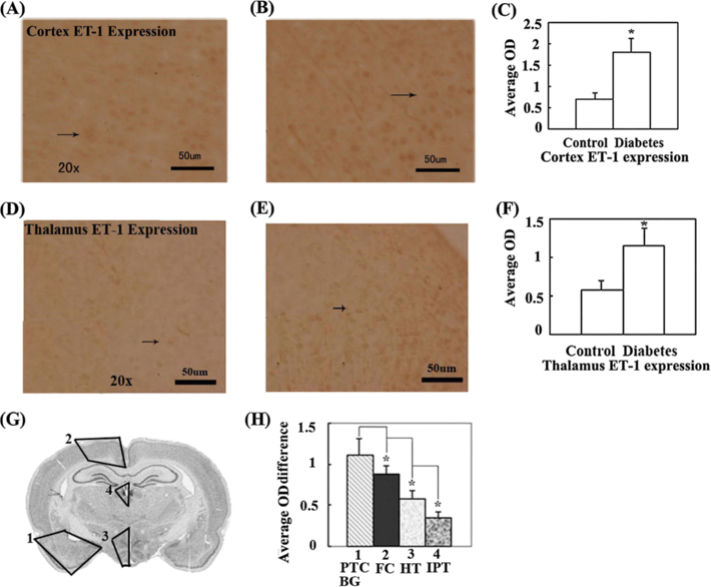
**ET-1 expression in distinct brain regions was compared by measuring the OD (Optic density) of positive cells in brains of control and diabetic rats with immunohistochemical staining. (A)**. The representative image of ET-1 expression in the cerebral cortex of control rats. **(B)**. The representative image of ET-1 expression in the cerebral cortex of diabetic rats. **(C)**. The OD comparison of ET-1 expression positive cells in cerebral cortex between control and diabetic rats. ET-1 expression in the cerebral cortex of diabetic rats significantly increases compared with control rats (*P < 0.05). **(D)**. The representative image of ET-1 expression in the thalamus of control rats. **(E)**. The representative image of ET-1 expression in the thalamus of diabetic rats. **(F)**.The OD comparison of ET-1 expression positive cells in thalamus between control and diabetes rats. ET-1 expression in the thalamus of diabetes rats significantly increases compared with control rats (*P < 0.05). **(G)**.Schematic representation indicates brain regions where ET-1 expression is significantly altered in diabetic rats and the rank order (from more to fewer) of expression quantity change in distinct regions of the integral brain. **(H)**.The comparison of ET-1 expression quantity change in distinct brain regions. ET-1 expression quantity change is significantly different in distinct brain regions of diabetic rats (*P < 0.05). The black arrow indicates the positive cell. The marked number in distinct regions of the rat brain atlas reflects the rank order (from more to fewer) of expression quantity change in distinct regions of integral brain in all figures. Bar = 50 μm. Abbr. Basal ganglia, BG; Frontal cortex, FC; Hippocampus, HI; Hypothalamus, HT; Inferior part of thalamus, IPT; Partial frontal cortex, PFC; Partial temporal cortex, PTC; Temporal cortex, TC; Thalamus, TH.

In the observed CVD susceptible brain regions, the ET-1 expression significantly increased in partial frontal and temporal cortex, basal ganglia, hypothalamus and the inferior part of thalamus (Figure 1A-H) in diabetic rats. The rank order (from more to fewer) of ET-1 expression quantity change in the diabetic rats showed that the partial temporal cortex and basal ganglia exhibited greater change than the frontal cortex. The frontal cortex exhibited greater change than the hypothalamus, which exhibited greater change than the inferior part of the thalamus (i.e., Partial temporal cortex and basal ganglia > frontal cortex > hypothalamus > inferior part of thalamus.) (Figure 1G, H). The regions with significantly altered ET-1 roughly included the amygdaloid nucleus (Amyg), posterior interposed nucleus (Pir), endopiriform nucleus (En), interstitial nucleus of posterior limb of anterior commissure (IPAC), nucleus of stria terminalis (BsTIA) and lateral globus pallidus (LGP) in the temporal cortex and basal ganglia; the retrosplenial agranular cortex (RSA), retrosplenial granular cortex (RSG), primary motor cortex (M1) and supplementary motor cortex (M2) in the frontal cortex; the dorsomedial nucleus of hypothalamus (DM), hypothalamic nucleus (HyT), supraoptic nucleus (SO), subincertal nucleus (SubI), zona incerta (ZI), xiphoid thalamic nucleus (Xi) and lateral hypothalamic area (LH) in the hypothalamus; and the paraventricular thalamic nucleus (PV), habenular nucleus (Hb) and mediodorsal thalamic nucleus (MD) in the inferior part of the thalamus.

### Expression of CGRP significantly decreased in the partial cerebral cortex and thalamus of diabetic rats

**Figure 2:**
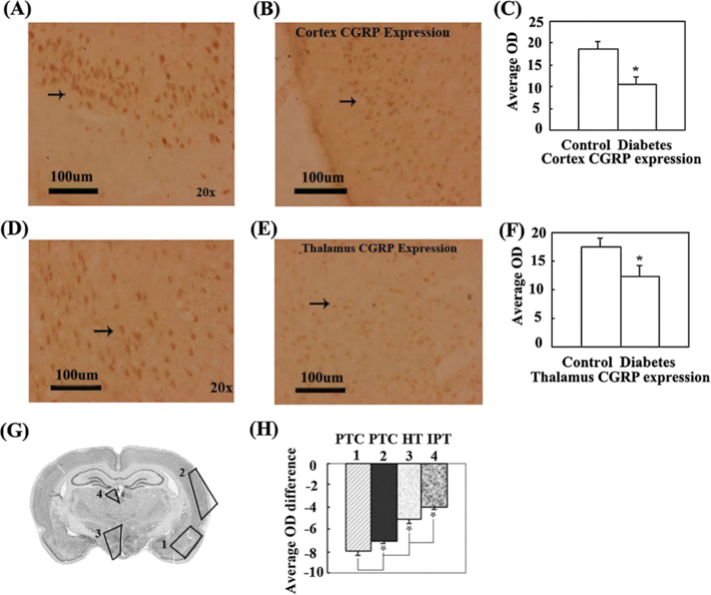
**CGRP expression in distinct brain regions were compared by measuring the OD of positive cells in brains of control and diabetic rats with immunohistochemical staining. (A)**. The representative image of CGRP expression in the cerebral cortex of control rats. **(B)**. The representative image of CGRP expression in the cerebral cortex of diabetic rats. **(C)**. The OD comparison of CGRP expression positive cells in cerebral cortex between control and diabetic rats. CGRP expression in the cerebral cortex of diabetic rats significantly decreases compared with control rats (*P < 0.05). **(D)**. The representative image of CGRP expression in the thalamus of control rats. **(E)**. The representative image of CGRP expression in the thalamus of diabetic rats. **(F)**. The OD comparison of CGRP expression positive cells in thalamus between control and diabetic rats. CGRP expression in the thalamus of diabetic rats significantly decreased compared with control rats (*P < 0.05). **(G)**. Schematic representation indicates brain regions where CGRP expression is significantly altered in diabetic rats and the rank order (from more to fewer) of expression quantity change in distinct regions of the integral brain. **(H)**. The comparison of CGRP expression quantity change in distinct brain regions. CGRP expression quantity change is significantly different in distinct brain regions of diabetic rats (*P < 0.05). Bar = 100 μm.

CGRP expression significantly decreased in the partial temporal cortex, hypothalamus and inferior part of the thalamus in diabetic rats (Figure 2A-H). The rank order of CGRP expression quantity change was partial temporal cortex > hypothalamus > inferior part of thalamus (Figure 2G, H), CGRP expression in the frontal cortex were not significant difference in the comparison of control and diabetic rats (No data shown). These significantly altered regions roughly consisted of Pir, En, Amyg, entorhinal cortex (Ent), perirhinal cortex (PRh) and ectorhinal cortex (Ect) in the temporal cortex; DM, HyT, SO, SubI, ZI, Xi and LH in the hypothalamus; and Hb, PV and MD in the inferior part of the thalamus.

### Expression of VCAM-1 significantly increased in the partial cerebral cortex, hypothalamus and hippocampus of diabetic rats

**Figure 3:**
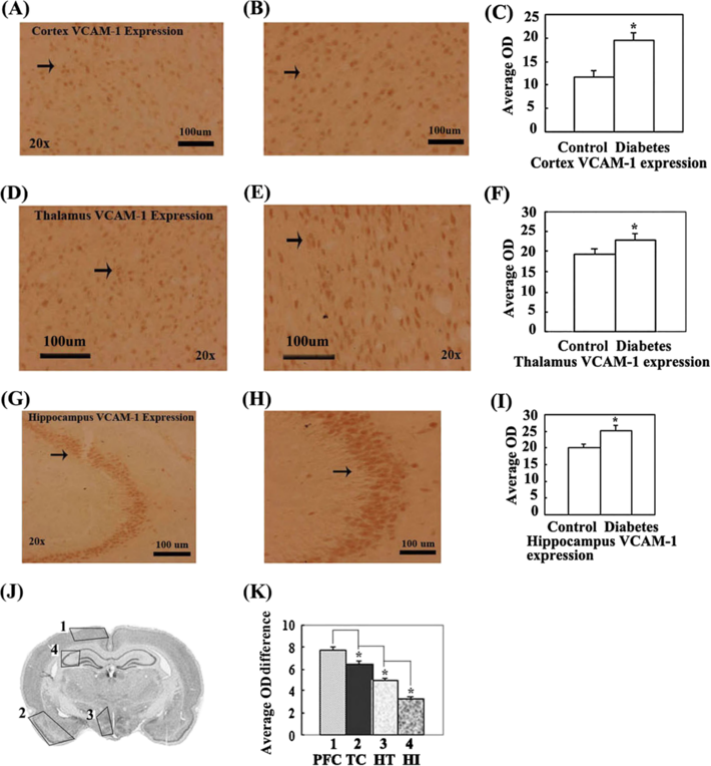
**VCAM-1 expression in distinct brain regions were compared by measuring the OD of positive cells in brains of control and diabetic rat with immunohistochemical staining. (A)**. The representative image of VCAM-1 expression in the cerebral cortex of control rats. **(B)**. The representative image of VCAM-1 expression in the cerebral cortex of diabetic rats. **(C)**. The OD comparison of VCAM-1 expression positive cell in cerebral cortex between control and diabetic rats. VCAM-1 expression in the cerebral cortex of diabetic rats significantly increases compared with control rats (*P < 0.05). **(D)**. The representative image of VCAM-1 expression in the thalamus of control rats. **(E)**. The representative image of VCAM-1 expression in the thalamus of diabetic rats. **(F)**. The OD comparison of VCAM-1 expression positive cells in thalamus between control and diabetic rats. VCAM-1 expression in the thalamus of diabetic rats significantly increases compared with control rats (*P < 0.05). **(G)**. The representative image of VCAM-1 expression in the hippocampus of control rats. **(H)**. The representative image of VCAM-1 expression in the hippocampus of diabetic rats. **(I)**. The OD comparison of VCAM-1 expression positive cells in hippocampus between control and diabetic rats. VCAM-1 expression in the hippocampus of diabetic rats significantly increases compared with control rats (*P < 0.05). **(J)**. Schematic representation indicates brain regions where VCAM-1 expression is significantly altered in diabetic rats and the rank order (from more to fewer) of expression quantity change in distinct regions of integral brain. **(K)**. The OD comparison of positive cells between control and diabetic rats; VCAM-1 expression significantly increases in the hippocampus of diabetic rats (*P < 0.05). Bar = 100 μm.

VCAM-1 expression significantly increased in partial frontal cortex, temporal cortex, hypothalamus and hippocampus in diabetic rats (Figure 3A-K). The rank order of VCAM-1 expression quantity change was partial frontal cortex > temporal cortex > hypothalamus > hippocampus (Figure 3J, K). These significantly altered regions were roughly constructed by M1, M2 and RSA in the frontal cortex; Pir, En, Amyg in the temporal cortex; DM, SubI in the hypothalamus; and CA2, CA3 in the hippocampus.

### Expression of ICAM-1 significantly increased in the partial cerebral cortex of diabetic rats

**Figure 4:**
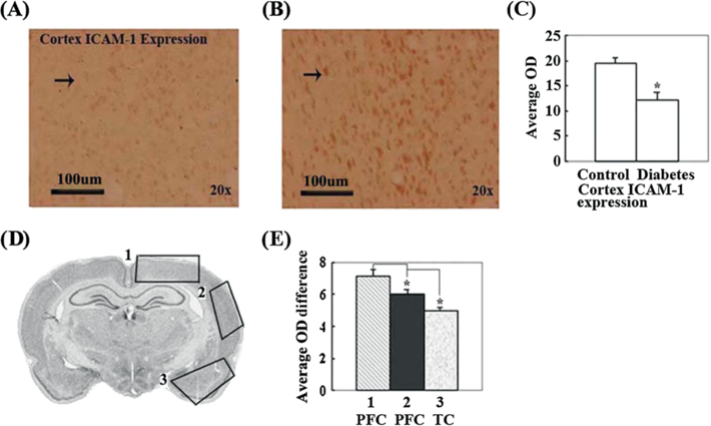
**ICAM-1 expression in distinct brain regions were compared by measuring the OD of positive cells in the brains of control and diabetic rat with immunohistochemical staining. (A)**. The representative image of ICAM-1 expression in the cerebral cortex of control rats. **(B)**. The representative image of ICAM-1 expression in the cerebral cortex of diabetic rats. **(C)**. The OD comparison of ICAM-1 expression positive cell in cerebral cortex between control and diabetes rats. ICAM-1 expression in the cerebral cortex of diabetic rats significantly increases compared with control rats (*P < 0.05). **(D)**. Schematic representation indicates brain regions where ICAM-1 expression is significantly altered in diabetic rats and the rank order (from more to fewer) of expression quantity change in distinct regions of the integral brain. **(E)**. The comparison of ICAM-1 expression quantity change in distinct brain regions. ICAM-1 expression quantity change is significantly different in distinct brain regions of diabetic rats (*P < 0.05). Bar = 100 μm.

ICAM-1 expression significantly increased in the partial frontal and temporal cortex in diabetic rats (Figure 4A-E). The rank order of ICAM-1 expression quantity change was partial frontal cortex > temporal cortex (Figure 4D, E). These significantly altered regions were roughly constructed by M1, M2 and RSA in the frontal cortex and by the second somatosensory cortex (S2), Ect, PRh, Ent, Pir, En and Amyg in the temporal cortex.

### Expression of P-selectin significantly increased in the partial cerebral cortex, basal ganglia, almost integral hippocampus and thalamus of diabetic rats

**Figure 5:**
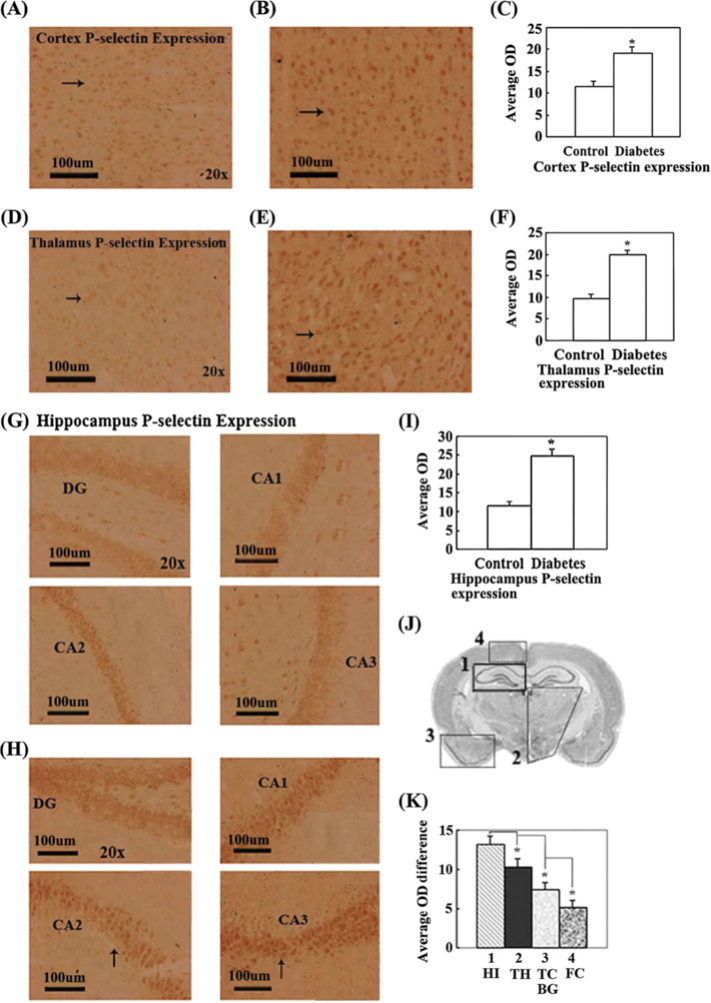
**P-selectin expression in distinct brain regions were compared by measuring the OD of positive cells in brains of control and diabetic rat with immunohistochemical staining. (A)**. The representative image of P-selectin expression in the cerebral cortex of control rats. **(B)**. The representative image of P-selectin expression in the cerebral cortex of diabetic rats. **(C)**. The OD comparison of P-selectin expression positive cell in cerebral cortex between control and diabetes rats. P-selectin expression in the cerebral cortex of diabetic rats significantly increases compared with control rats (*P < 0.05). **(D)**. The representative image of P-selectin expression in the thalamus of control rats. **(E)**. The representative image of P-selectin expression in the thalamus of diabetic rats. **(F)**. The OD comparison of P-selectin expression positive cells in thalamus between control and diabetic rats. P-selectin expression in the thalamus of diabetic rats significantly increases compared with control rats (*P < 0.05). **(G)**. The representative image of P-selectin expression in the hippocampus of control rats. **(H)**. The representative image of P-selectin expression in the hippocampus of diabetic rats. **(I)**. The OD comparison of P-selectin expression positive cells in hippocampus between control and diabetic rats. P-selectin expression in the hippocampus of diabetic rats significantly increases compared with control rats (*P < 0.05). **(J)**. Schematic representation indicates brain regions where P-selectin expression is significantly altered in diabetic rats and the rank order (from more to fewer) of expression quantity change in distinct regions of the integral brain. **(K)**. The comparison of P-selectin expression quantity change in distinct brain regions. P-selectin expression quantity change is significantly different in distinct brain regions of diabetic rats (*P < 0.05). Bar = 100 μm.

P-selectin expression significantly increased in the partial frontal and temporal cerebral cortex, almost integral hippocampus and thalamus in diabetic rats (Figure 5A-K). The rank order of ICAM-1 expression quantity change was hippocampus > thalamus > temporal cortex and basal ganglia > frontal cortex (Figure 5J, K). These significantly altered regions roughly included the dentate gyrus (DG), CA1, CA2 and CA3 in hippocampus; almost all nuclei in the thalamus; Amyg, Pir, En, IPAC, BsTIA and LGP in the temporal cortex and basal ganglia; and M1, M2, RSA and RSG in the frontal cortex.

## Discussion

In this study, rats consistent with the characteristics of a diabetic rat model and scores of 0 based on Longa’s standard were selected as the diabetic rat model of stroke prophase. ET-1, CGRP, VCAM-1, ICAM-1 and P-selectin expression was semi-quantitatively analyzed by immunohistochemical staining and the measurement of OD of immunohistochemical positive cells. The positions of various brain regions were located using an atlas (The Rat Brain in Stereotaxic Coordinates, Sixth Edition: Hard Cover Edition). The expression changes of endothelium-associated factors/vasoactivators, including ET-1, CGRP, VCAM-1, ICAM-1 and P-selectin, were compared and analyzed in CVD-susceptible brain regions such as the frontal and temporal cortex, basal ganglia and thalamus.

The results of this study provide several lines of evidence implicating CVD in T2DM. First, these data demonstrate that the expression of ET-1, VCAM-1, ICAM-1, P-selectin and CGRP is significantly altered (increased or decreased) in multiple brain regions, including the partial frontal and temporal cerebral cortex, basal ganglia, hippocampus and thalamus, in diabetic rats at 4 weeks post-induction of diabetes. These described brain regions are the susceptible brain regions of CVD; therefore, the data indicate that cerebral vascular complications in T2DM are associated with alterations of these endothelial function-associated factors. Second, our results contribute to a growing body of evidence indicating that the ETD contributes to CVD in T2DM by an imbalance of ET-1, CGRP, VCAM-1, ICAM-1 and P-selectin expression, because the imbalance of these endothelial function-associated factors contributes to ETD responses. The data also support that alterations of ET-1, CGRP, VCAM-1, ICAM-1 and P-selectin expression play a role in CVD complications in T2DM via ETD. Third, the significant expression alterations of ET-1, CGRP, VCAM-1, ICAM-1 and P-selectin occurs in emotion center (partial temporal cortex and hippocampus of the limbic lobe), cerebral splanchno-motor center (partial M1, M2 and RSA in the frontal cortex), and neuroendocrine center (partial nuclei in the hypothalamus and inferior thalamus), which might directly or indirectly mediate expression of these factors in T2DM via the central nervous system. Furthermore, the abnormal brain vascular constriction and vasodilation caused by an imbalance of ET-1 and CGRP expression and the increase in brain vascular permeability induced by abnormal expression of VCAM-1, ICAM-1 and P-selectin participate in CVD complications in T2DM by ETD, which provides new evidence for preventing the related CVD in diabetes. In addition, there is a significant difference in the expression of studied vasoactivators in the different brain regions of diabetic rats. This may indicate that the significant altered regions might play a more important role in the modulation and function of the individual vasoactivator.

Although the pathogenesis of ETD is not completely clear, it is generally accepted that ETD is caused by an imbalance between endothelium-derived vasodilator and vasoconstrictor substances. ET-1 is a potent vasoconstrictor produced by endothelial cells (EC). The production and the plasma levels of ET-1 are elevated in patients with diabetes, and a positive correlation between plasma ET-1 levels and diabetic microangiopathy has been reported. Thus, it is possible that ET-1 plays a potential role in ETD by an imbalance between endothelium-derived vasodilator and vasoconstrictor substances on the pathophysiology of vascular complications in diabetes [9].

In this study, we studied ET-1 expression in the susceptible brain regions of CVD in a rat model of T2MD and compared it with the expression in controls. It was found that ET-1 expression was significantly elevated in the partial frontal and temporal cortex, basal ganglia, hypothalamus and inferior part of the thalamus in diabetic rats. These brain regions are susceptible to atherosclerosis and CVD (See Figure 1A-H). The exact mechanism by which ET-1 causes CVD and/or ETD in T2DM is not completely known yet; however, there are deleterious effects on the studied brain regions causing EC damage and increased local vasoconstriction of these brain regions, ultimately leading to brain vascular damage such as atherosclerosis and chronic brain ETD. In addition to its direct vasoconstrictor effects, enhanced levels of ET-1 may contribute to ETD through the production of a series of vascular active factors, including ROS (reactive oxygen species), NO and inflammatory factors [10-12].

CGRP is an important moderator of ET-1 vasoconstriction. The decrease in CGRP expression results in an imbalance in the CGRP/ET-1 ratio, which induces abnormal vasoconstriction to contribute to direct and/or indirect local ETD and vascular damage [13,14]. In our results, diabetes induced a significantly decreased immunoreactivity of CGRP in the partial temporal cortex, hypothalamus and inferior part of the thalamus (See Figure 2A-H), which indicates that diabetes contributes to decreases CGRP expression within the CVD susceptible regions. The markedly decreased CGRP expression in the distinct brain regions of rats subjected to diabetes results in an imbalance of CGRP and ET-1 content. Therefore, we suggests that an imbalance of CGRP and ET-1 content in CVD-susceptible regions might take part in the development of CVD in T2DM via ETD.

VCAM-1 is one of the major endothelial receptors that mediate leukocyte adhesion to the vascular endothelium. Recent data have strongly suggested that VCAM-1 may play an important role in the pathogenesis of atherosclerosis because VCAM-1 function in leukocyte adhesion and transmigration is crucial and its expression is induced early in nascent atheroma plaques [15]. In addition, VCAM-1 expression is induced by proinflammatory stimuli, including TNF-α and IL-1β, and is mediated, at least in part, by NF-kb [16]. VCAM-1 expression is also induced under vascular stress conditions such as insulin resistance and chronic hyperglycaemia. Under these conditions, an increase in VCAM-1 expression activity was also observed [17,18]. In addition to binding leukocytes, VCAM-1 engagement contributes to leukocyte transendothelial migration (TEM) by stimulating gap formation between cells in the endothelial monolayer, which could facilitate TEM [19]. This gap formation is mediated by VCAM-1 [20,21]. VCAM-1 clustering also increases the intracellular free calcium concentration [20]. Thus, VCAM-1 expression increases vascular endothelium permeability and leukocyte TEM, contributes to vascular damage and ETD, and induces the generation of atherosclerosis.

The endothelial expression of ICAM-1 is increased in atherosclerosis and in animal models of atherosclerosis [22,23]. ICAM-1 is normally present in low levels on ECs, but its expression is dramatically increased in response to proinflammatory stimuli, including the proinflammatory cytokines TNF-α, IL-1β, and interferon-γ [23]. Soluble ICAM-1 may modulate leukocyte adhesion and migration. Facilitating leukocyte attachment to the endothelial surface is not the only function of ICAM-1. Engagement of ICAM-1 also induces signaling in ECs, increases the production of IL-8 and induces expression of ICAM-1 and c-fos. It is possible that proinflammatory IL-8 and c-fos activation by ICAM-1 initiates a positive feedback loop, leading to more ICAM-1 and VCAM-1 expression and thereby promoting the sustained recruitment of leukocytes to areas of atherosclerosis, accelerating the process of atherosclerosis by proinflammatory stimuli via indirect or direct ETD [24-26].

P-selectin is also regulated by proinflammatory stimuli, which is stored in intracellular vesicles of ECs that fuse with the plasma membrane in response to a number of stimuli, including ischemia and chronic hyperglycaemia [27]. In some circumstances, P-selectin translocation to the EC surface is regulated through a ROS-dependent mechanism. P-selectin clustering increases cytosolic free calcium and induces changes in cell morphology, promoting ETD to consequently produce vascular damage [28-30].

As described above, the previous research reflected the expression, regulation and function of the three adhesion molecules (ICAM-1, VCAM-1 and P-selectin). First, there is evidence that expression of the soluble adhesion molecules VCAM-1, ICAM-1 and P-selectin is associated with proinflammatory stimuli. Elevated levels of endothelial adhesion molecules (EAM) may, to some extent, reflect a chronic inflammatory state, because systemic inflammation can directly elicit overproduction of EAM [31]. Second, the atherosclerotic process appears to be associated with alterations in adhesion molecules of endothelial function-associated vasoactivators because VCAM-1, ICAM-1 and P-selectin play an important role in vascular integrity and permeability via ETD [32]. Elevated levels of soluble EAM may be one of the common antecedents for the pathogenesis of both atherosclerotic CVD and ETD [32]. The expression of endothelial cellular adhesion molecules promotes the adherence and transmigration of leukocytes into the subendothelial space, eventually leading to ETD and subendothelial structural changes [33]. Increased vascular permeability due to structural alterations can then reduce insulin delivery to insulin-sensitive peripheral tissues, which in turn produces insulin resistance. Alternatively, insulin resistance may directly promote ETD [34]. The study in nondiabetic individuals have suggested that mildly impaired glucose tolerance within the normoglycemic range may accelerate the progression of ETD via adverse effects on oxidative stress, formation of advanced glycation end products, and elevated levels of free fatty acids [35]. Vascular ETD may precede insulin resistance, induce a reduction in insulin sensitivity, and produce a malignant cycle [36-38].

Our results show a significant increase in VCAM-1, ICAM-1 and P-selectin in CVD susceptible brain regions of T2DM rats (see Figures 3, 4 and 5). The results also provide experimental evidence of diabetic angiopathy, which is generated by ETD through increases in VCAM-1, ICAM-1 and P-selectin. The results also indicate that diabetes can elicit the expression of adhesion molecules, including P-selectin, ICAM-1, and VCAM-1, which indirectly or directly promote development of ETD to induce CVD.

Our study described alterations of ET-1, CGRP, VCAM-1, ICAM-1 and P-selectin expression in main CVD susceptible brain regions of T2DM rats (see Figures 1, 2, 3, 4 and 5). These findings highlight the therapeutic potential of targeting the vascular endothelium activators for prevention and/or treatment of CVD disease in T2DM. Of note, there is a growing body of evidence on interventions aimed at mediating individual risk factors correlated with ETD, such as ET-1, CGRP, VCAM-1, ICAM-1 and P-selectin. Our studies also support a link between ETD and T2MD, implicating a role for ETD in the CVD pathogenesis of T2DM. To date, very few studies have directly evaluated the alterations of ET-1, CGRP, VCAM-1, ICAM-1 and P-selectin in CVD-susceptible brain regions of a T2DM model. Here, we report that significant expression alterations of ET-1, CGRP, VCAM-1, ICAM-1 and P-selectin occurred in CVD susceptible brain regions in T2DM rats, including the frontal and temporal cerebral cortex, basal ganglia and thalamus.

## Conclusions

Our results offered the more evidences that ETD plays an etiological role in the pathogenesis of CVD in T2DM, and also gives new insights into the potential clinical value of endothelial function modulation in CVD of T2DM. The pathogenesis of CVD in T2DM might be associated with an expression imbalance of ET-1, CGRP, VCAM-1, ICAM-1 and P-selectin, which promotes ETD through a series of chemical factors, such as ROS, NO and inflammatory factors. In addition, it was suggest that emotion, cerebral splanchno-motor and neuroendocrine center might be involved in the pathogenesis of CVD in T2MD via expression alterations of ET-1, CGRP, VCAM-1, ICAM-1 and P-selectin. However, further studies are warranted.

## Abbreviations

ANG-II: Angiotensin II; CGRP: Calcitonin gene related peptide; ETD: Endothelial dysfunction; ET-1: Endothelin-1; ICAM: Intercellular adhesion molecule; LCAM: Leukocyte adhesion molecule; NO: Nitric oxide; PAI-1: Plasminogen activator inhibitor-1; STZ: Streptozotocin citric acid; t-PA: Tissue-type plasminogen activator; VCAM: Vascular cell adhesion molecule; Vwf: Von Willebrand factor.

## Competing interests

The authors declare that they have no competing interests.

## Authors’ contributions

XR, WH, and WY conceived and designed the experiments, YR, HH, and XQ performed the experiments, XR, YR, HH, and XQ analyzed the data, contributed reagents/materials/analysis tools, XR, YR, HH, and XQ wrote the paper. All authors read and approved the final manuscript.
